# Methodological reflections to support good practice in using nominal group techniques: Insights from applications in palliative care studies

**DOI:** 10.1177/02692163251368974

**Published:** 2025-09-28

**Authors:** Hui-Ju Liang, Qian Xiong, Nancy Preston

**Affiliations:** 1 Division of Health Research, Faculty of Health and Medicine, Lancaster University, Lancaster, UK; 2 Centre for Ageing Research, Division of Health Research, Faculty of Health and Medicine, Lancaster University, UK; 3 International Observatory on End of Life Care, Division of Health Research, Lancaster University, UK

**Keywords:** Nominal group technique, consensus methods, recommendation development, interpretive qualitative analysis, framework analysis

## Abstract

**Background:**

High-quality recommendations require rigorous methods based on strong evidence to improve clinical practice. In palliative and end-of-life care, expert consensus is sometimes achieved through nominal group techniques. However, its practical challenges are often underestimated, potentially compromising the rigour and the quality of the recommendations.

**Methodological reflections:**

The methodological reflections on developing recommendations using the nominal group technique are discussed in this paper. These reflections are drawn from its theoretical foundations and applications in palliative care research, including a Taiwanese study on preparing families for a relative’s death. We highlight key issues such as the omission of pilot meetings and the underestimation of practical challenges in conducting group meetings, including time constraints and real-world uncertainties, especially during the stages of listing, clarifying, voting and ranking recommendations. Cultural factors are often overlooked, as seen in the example study, where the moderator avoided interruptions to show respect and politeness during the meeting. Additionally, valuable data gathered during meetings is often underutilised. These factors collectively can undermine the quality of recommendations. Based on these insights, we offer suggestions for improvement.

**Key suggestions:**

Pilot meetings should be conducted and reported to demonstrate how they inform the main meeting, ensuring research rigour and recommendation quality. Sufficient time should be allocated for listing and clarifying recommendations and in societies with specific etiquettes (e.g. minimising interruptions to show politeness). Further qualitative analysis of meeting transcripts is suggested to better understand the context and rationale behind the recommendations and enhance their applicability and clarity.

**What is already known about the topic?**

- Developing practice recommendations based on evidence using rigorous and transparent methods is essential for enhancing healthcare quality through formal consensus methods such as the nominal group technique.
- The nominal group technique is a structured method for identifying priorities, reaching consensus and developing recommendations, but despite its wide use in healthcare research, it remains underutilised in palliative care.

**What this paper adds?**

- We reinforce the value of conducting pilot meetings and highlight the practical challenges of conducting nominal group technique meetings, as difficulties arise from multiple tasks, time constraints, practical complexities and real-world uncertainties, particularly during the recommendation listing, clarification, voting and ranking phases.
- Cultural factors in applying the nominal group technique are overlooked, as shown in the example study, including the need for more time to summarise and record participants’ verbal recommendations in written Traditional Chinese (which uses complex characters with many strokes) and the necessity of adhering to etiquette by minimising interruptions to demonstrate politeness.
- Existing palliative care research using nominal group techniques often underutilises valuable qualitative data from group meetings, overlooking opportunities to reflect on their conduct or address practical challenges that have been encountered during the process through post-meeting qualitative analysis.

**Implications for practice, theory or policy**

- Pilot meetings should be conducted and reported to show how they inform the main meeting, ensuring research rigour to improve recommendation quality.
- Sufficient time should be allocated for conducting nominal group technique meetings, particularly for listing and clarifying recommendations, with culturally appropriate consideration given to societies where interruptions are avoided to show politeness, necessitating prior arrangements made with participants.
- Further qualitative analysis of group meeting transcripts through a more interpretive qualitative approach (e.g. framework analysis), particularly of discussion during recommendation listing and clarification, is recommended to reflect on meeting conduct, gain deeper insights into the context and rationale behind participant-generated recommendations, and enhance their applicability and practicability.

## Background

Implementing research findings into clinical practice is essential for enhancing patient care and maintaining healthcare quality.^1–3^ The quality of recommendations depends on the strength of the underlying evidence.^1^ In palliative and end-of-life care research, expert consensus together with engaging practitioners in the stage of developing evidence-based recommendations is vital.^2–4^ Formal consensus methods, such as the Delphi method and nominal group technique, are commonly used to establish consensus and involve practitioners in the process.^5–9^

The nominal group technique, developed in the 1970s, is a structured method for generating recommendations that prioritise group consensus over diverse perspectives.^8,10–12^ Unlike the multi-round Delphi method, it is more time-efficient and less demanding for participants, requiring only a single session.^5^ This method ensures equal participation opportunities to contribute,^5,11^ preventing domination by certain individuals, a common issue in focus groups.^13,14^ The technique comprises four stages: preparation, sampling, group meeting and data analysis^5,11,12,15^ (Table 1). The preparation stage involves practical tasks such as planning the meeting, setting its duration, and arranging space and equipment, alongside research-related tasks like gathering relevant evidence^5,7,9^ and conducting pilot meetings.^5^ In the sampling stage, participants with diverse expertise are selected, usually through purposive sampling,^5,9,12^ to enhance data quality and minimise social hierarchy effects.^5^ Nominal group meetings typically involve five to ten participants, depending on the research scope and participants’ availability, ensuring adequate data collection while providing equal opportunities for all participants to contribute.^5,9,15^

**Table 1.** Process of the nominal group technique.^5,6,11^

Stages	Main tasks
Preparation	Organising the meeting procedures, such as scheduling and securing meeting space and equipment. Preparing a synthesis of evidence on research topics to be presented to participants. Conducting a pilot meeting to test the meeting procedures and research questions to be presented during the meeting.
Sampling	Recruiting participants based on the study’s inclusion and exclusion criteria.
Running group meeting	The meeting procedure involves an introduction, presentation of relevant research evidence, silent recommendation generation, recommendation listing and clarification, voting and ranking, and conclusion.
Data analysis	Data from nominal group technique meetings, including recommendation counts, recommendation lists, and voting and ranking results, is typically analysed using quantitative and qualitative methods.

Group meetings are the core of the nominal group technique, following a structured process of introduction, recommendation generation, listing, voting and ranking.^5,11^ The moderator begins by outlining objectives and expected outcomes, followed by brief participant introductions.^11,15^ Relevant research evidence is presented before introducing the meeting’s research questions.^5,16,17^ Participants then generate recommendations individually and silently, without discussion.^5,11,12^ Next, they take turns sharing one recommendation at a time, trying to avoid repetition while promoting equal participation and diverse input.^12,15,18^ A discussion phase follows for clarification before voting and ranking to reach consensus.^11,15^ The meeting concludes with a summary of key outcomes, expressions of gratitude and final questions.^5^ Meetings typically last 90–120 min,^5,15^ depending on group size, the number of research questions and available time.^18^ Two moderators usually lead the session, with one facilitating and the other observing and taking notes. Collected data, including recommendation lists, counts and voting results, are analysed using quantitative and qualitative methods.^5,15^

The nominal group technique serves multiple research purposes, including developing recommendations, solving problems, establishing priorities and building consensus.^5,8,19^ It is suited to explore sensitive or under-researched topics,^5^ which are common in palliative care. Although widely applied in healthcare and nursing,^6^ its application in palliative care research remains limited.^20^ This paper aims to provide methodological reflections on the technique, drawing from its theoretical foundations and applications in selected exemplar palliative care studies. While focused on this field, the insights may be relevant more broadly.

Relevant literature was identified through Scopus and Google Scholar using the keywords ‘nominal group technique’ and ‘palliative care’. Sixteen papers published between 2006 and 2024 were selected based on relevance to palliative care.^16,21–35^ A summary of how the method has been used is available in the supplementary file (S1). Collectively, these studies demonstrate the technique’s adaptability. It has been used to explore research conduct.^24,26^ research priorities,^16^ clinical practice,^28,31,32^ outcome measurement^29,33,35^ and professional education.^23^ Many studies included participants in varied roles,^16,21,24–33,35^ such as patients, family members and healthcare professionals,^24,35^ demonstrating the technique’s capacity to capture diverse perspectives. Some studies combined the technique with other research methods.^21,24,28^ Seven studies presented relevant evidence during meetings,^16,24–29^ which can enhance recommendation quality, participant engagement and group cohesion.^5,7^ However, nine studies omitted this step,^21–23,30–35^ possibly to emphasise participants’ lived experiences, underscoring the method’s flexibility.

Challenges in applying the nominal group technique were reported in 11 studies (see Table 2 for details), including participant recruitment,^21,22,24,26–29,35^ the conduct of the meeting,^24,34,35^ reaching consensus,^33^ data analysis,^24,34,35^ and verification of recommendations with participants after the meeting.^23,26^ In contrast, five studies did not state any challenges.^16,25,30–32^ Overlooking these issues can compromise methodological rigour and the quality of recommendations produced. These challenges are further reflected upon in relation to our study, as discussed below.

**Table 2.** Challenges reported in exemplar palliative and end-of-life studies using the nominal group technique^a^.

Authors, year, country	Challenges encountered in practice					Key
	Participant- related	Conduct of meeting	Establish consensus	Data analysis	Participant rechecking	
Aspinal et al.^35^, UK		1		2		1 = Research question potentially interpreted differently across groups 2 = Analysing data across groups 3 = Challenges in grouping and ranking for participants with limited literacy 4 = Diverse recommendations made consensus difficult. 5 = Limited participant diversity – hard to get non-health care professionals 6 = Limited time to conduct meeting to reach consensus 7 = Limited time to create clear recommendations 8 = Difficulty analysing recommendations that had multiple parts 9 = Findings not presented to participants for feedback
Tuffrey-Wijne et al.^34^, UK	3			2		
Pastrana et al.^33^, Germany			4			
Higginson et al., 2013, UK ^24^	5	6,7		8		
Rice et al.^22^, USA, Canada	5					
de Wolf-Linder et al.^29^, Ireland	5					
Walshe et al.^28^, UK	5					
Dhingra et al.^27^, 2022, USA	5					
Hussain et al.^26^, 2022, UK	5				9	
Hökkä et al.^23^, Europe					9	
Walshe et al.^21^, UK	5					

a Five of 16 studies did not report any challenges.^16,25,30–32^ UK: United Kingdom, USA: United States.

## A practical example of the nominal group technique

The example study is part of a larger project aimed at improving the preparation for a relative’s death in Taiwan^36–38^ underpinned by critical realism, acknowledging that truth is contextualised ^39^ This study aimed at developing recommendations for healthcare professionals on preparing families for a relative’s death using the nominal group technique. On 8th July 2023, a nominal group meeting was held in Taiwan with ten specialist palliative care professionals, including two physicians, six nurses, one psychologist, and one chaplain. The participants generated and voted upon 42 recommendations, resulting in a prioritised list.

## Methodological reflections on the nominal group technique

The real-world challenges of applying the nominal group technique are explored, addressing both its theoretical foundations and practical applications in palliative care research.

### The absence of pilot meetings or poor reporting

Pilot meetings are recommended to refine meeting procedures, improve the clarity of research questions to be presented and enhance moderator confidence.^5,12,15,40^ By addressing potential challenges in advance, they help ensure smoother main sessions and improve recommendation quality.^5,9,40^ For methodological rigour and transparency, changes made following pilot findings and their impact on the formal session should be reported. However, pilot meetings were often omitted or poorly documented in the exemplar palliative care studies.^16,21,24–35^ Only two studies reported conducting pilot meetings.^22,23^ One found no relevant outcomes,^23^ while the other extended discussion time but made no protocol changes.^22^ However, the pilot involved research team members rather than intended participants (family caregivers) and lacked details on the original or revised discussion duration, limiting transparency.

In contrast, our study reinforced the value of pilot meetings by providing feedback that refined the main session. The pilot, held on 8 June 2023 with 10 specialist palliative care nurses who did not participate in the main meeting, tested all procedures and led to key modifications. While recommendations are usually grouped into themes during the clarification stage^18^ or in post-meeting analysis,^15,35^ pilot feedback suggested introducing predefined themes, such as clinical care and education, during the listing stage. This helped identify recurring recommendations and streamline voting and ranking. Other refinements included sharing meeting objectives, procedures and research questions with participants in advance to allow for preparation and questions beforehand. Typing recommendations into a laptop and using voting software to improve efficiency and reduce manual errors was also suggested. Recognising the need for sufficient time, the meeting duration was extended from 140–150 min to allow more time for presenting evidence and listing and clarifying recommendations while slightly reducing the time for voting and ranking.

### Lack of clarity in defining group consensus

Establishing group consensus is a central goal of the nominal group technique. For methodological rigour, researchers should clearly define what constitutes consensus, including how it will be identified and measured.^5^ Theoretically, consensus refers to the degree of individual or overall group agreement and whether the collective views demonstrate sufficient alignment.^5^ In practice, consensus is typically reached through independent voting and ranking, followed by aggregation of results without further participant comments.^11^ Additional voting rounds may be conducted if needed.^5^ Importantly, verifying that participants accept the final outcomes confirms whether consensus has been achieved. This poses a methodological challenge in studies involving multiple groups, where researchers must decide whether to assess consensus within each group or across all groups collectively.

In the exemplar palliative care studies, definitions and processes for reaching consensus varied in rigour and transparency. Some followed clear procedures,^24,25^ while others lacked sufficient detail.^16,22,23,27,29–32,34,35^ Most commonly, consensus was achieved by selecting the top five^27,29–32,34,35^ or top ten^16,21,23^ recommendations after a single one voting round. However, few studies reported whether participant feedback was sought or whether additional rounds were conducted to confirm agreement. Only one study explicitly stated that consensus was reached when participants had no further comments and results were finalised ^25^ while another defined consensus as full participant agreement during the clarification phase rather than the ranking process.^22^ Moreover, multi-group studies rarely described how consensus was established across groups.^22,23,27,31,34,35^ A few used follow-up online surveys to assess cross-group consensus.^17,24,32,41,42^ Some used statistical measures such as median score and interquartile ranges,^17,24,41,42^ while another asked participants from multiple groups to prioritise the top five recommendations based on perceived importance.^32^ However, these approaches seldom addressed whether any disagreement remained, raising concerns about the robustness of consensus verification. Follow-up surveys may offer a practical solution for assessing consensus across groups.

In our example study, 42 recommendations were voted upon, and the 10 receiving the highest number of votes were prioritised. As the results were accepted by all participants, no additional voting rounds were required.

Overall, the lack of consistent definition and transparent reporting criteria for establishing consensus can compromise the reliability of nominal group technique studies. Clear operationalised definitions and explicit procedures for establishing consensus are essential for ensuring methodological rigour.

### Underestimate the practical challenges of conducting group meetings

Practical challenges in conducting nominal group meetings are often underestimated, yet they can undermine the rigour of the method and the quality of recommendations developed.^9^ One possible reason for this may be the technique’s inherent flexibility. Although theoretically structured, the nominal group technique varies in formats, research objectives and participant needs.^6,18,35,43^ Meetings may be conducted in person or online^6^ and may involve a single session or multiple sessions with different groups,^8,12,17,31,35^ held either simultaneously^12,17^ or asynchronously.^8,31,35^ Standard procedures involve listing recommendations without discussion, grouping similar ones and prioritising them through ranking and votes.^5^ However, the procedure of generating recommendations can be different. While recommendations are often written during meetings,^35^ some studies asked participants to prepare them in advance.^8,19^ Some adaptations allow discussion during the listing phase or grouping recommendations into broader themes during meetings.^18,40,43^ This adaptability is evident in the exemplar palliative care studies. Some conducted one in-person meeting^25,30,33^; others organised multiple in-person sessions for different groups, either simultaneously^16,24,26,29^ or asynchronously.^22,27,28,31,32,34,35^ Some opted for multiple online sessions for separate groups at different times.^21,23^ These format and timing choices reflect efforts to accommodate participants while maintaining the integrity of the consensus process. However, most studies did not report challenges encountered during meetings.^16,21–23,25–33^ Only a few noted specific difficulties,^24,35^ such as applying the same research question to different types of participants.^35^

The structured design and practical flexibility of the nominal group technique are strengths, but they do not eliminate implementation challenges.^18,43^ In our example study, despite a pilot meeting conducted, several issues arose during recommendation listing, clarification, voting and ranking. These challenges stemmed from managing multiple tasks under time pressure, linguistic complexity and real-world uncertainties. While recommendations should be recorded as accurately as possible to preserve participants’ original wording,^12,15^ it was difficult in practice. None of the exemplar palliative care studies reported challenges with capturing participants’ voices,^16,21–35^ but in our case, recording participants’ verbal recommendations in written Traditional Chinese, a language with complex characters and many strokes, within time constraints was challenging. This increased time pressure also raised the risk of human errors in subsequent voting and ranking stages. In our study, eight recommendations tied in voting, but three of the tied ones were missed in the final listing of the top ten recommendations.

Technologies, such as laptops, projectors and voting software, can streamline the meeting process and reduce manual errors.^21–23,31^ However, technical difficulties may still arise. In our case, typing handwritten recommendations in Traditional Chinese for voting software was more time-consuming than in English, increasing the likelihood of errors. Additionally, Mentimeter was planned to be used for ranking, but it failed during the meeting despite prior testing, so we had to switch to paper-based ranking.

Our study illustrates that practical challenges of conducting nominal group meetings can occur even after pilot meetings. Greater attention to these real-world barriers is essential to ensure robust application of the nominal group technique.

### Insufficient attention to cultural contexts

Cultural context is often overlooked in applying the nominal group technique.^43^ This limitation is apparent in the exemplar palliative care studies, likely due to studies predominately being conducted in Western societies, including the United Kingdom,^16,17,21,24–26,28,32,34,35,41,42^ Europe,^23,29–31,33^ the United States^27^ and Canada.^22^ Our study underscores the importance of considering cultural factors when applying the nominal group technique,^43^ particularly in non-Western contexts, to ensure both methodological rigour and cultural sensitivity.

In our example study conducted in Taiwan, cultural and linguistic factors affected the recommendation listing stage and prolonged the session. Summarising participants’ verbal recommendations during the listing stage was challenging. Participants struggled to make succinct suggestions, often presenting multiple recommendations at once, and had difficulty articulating their thoughts on death preparation. These patterns may reflect communication styles within Taiwanese culture.^44^ Consequently, the moderator needed to interpret participants’ statements, identify key points being made and confirm these with them. Despite awareness of time constraints, the moderator did not necessarily interrupt participants to manage the session, as avoiding interruptions to show respect and politeness is a cultural norm in Taiwan.^44^ Additionally, writing recommendations in Traditional Chinese slowed down the real-time documentation process. Therefore, much more time was spent on listing, clarifying and discussing recommendations than expected; our entire session still exceeded the adjusted duration (150 min) based on a pilot by approximately 80 min.

### Underutilisation of the rich qualitative data from group meetings

A reductionist approach, consolidating similar recommendations during meetings to minimise post-meeting analysis, is often used in the data analysis of the nominal group technique,^10^ leading to methodological challenges.^12^ Consequently, greater emphasis is placed on quantitative data for voting and ranking outcomes and cross-group comparisons.^43^ In contrast, qualitative analysis tends to be inconsistently applied and usually limited to the synthesis of participant-generated recommendations.^17^ In-depth analysis of meeting transcripts is rare and often poorly reported,^45^ leading to underutilisation of rich qualitative data from group meetings and loss of contextual insights into how recommendations are developed.^15,43^

This pattern is reflected in the exemplar palliative care studies. Voting and ranking results were typically analysed using descriptive statistics and compared across groups when needed.^22,27,31,34,35^ Qualitative data, including participant-generated recommendations,^17,22,23,28,31,35^ scribe notes^26,29^ and meeting transcripts,^25,27,32,33^ were analysed less consistently. Most studies focused on synthesising recommendations or scribe notes, often neglecting deeper insights available in transcripts.^22,23,26,28,29,31,35^ Thematic analysis was commonly used^23,25–29,33,35^, while some studies applied content analysis,^22,32^ or did not specify the method.^17,24,31^. A few studies omitted post-meeting qualitative analysis,^16,30,34^ possibly due to using reductionist approaches during meetings, which reduced the need for further analysis.^10^

While some exemplar palliative care studies analysed transcripts,^25,27,32,33^ few reflected on how meetings were conducted or practical challenges encountered. In our example study, challenges arose despite prior piloting. Although the moderator aimed to remain neutral,^40,45^ interactions during the recommendation listing and clarification stages proved complex. Time constraints and data complexity contributed to errors in voting and ranking, with recommendations made on the day missing important information from deeper discussions. This highlighted the need to critically examine the conduct of group meetings and the researcher’s role. An interpretive qualitative approach can offer greater insight into the context and reasoning behind participants’ recommendations and enhance the relevance and applicability of findings.

In our study, framework analysis was chosen to allow structured interpretation within a predefined theme framework.^46,47^ The process followed five stages: familiarisation, framework development, indexing, charting, and mapping and interpretation.^47^ The meeting recording was transcribed and reviewed to familiarise the researcher with the data. A framework was developed that included the recommendations listed and agreed upon by participants, along with the top 10 ranked recommendations. It also incorporated summaries of discussions about each recommendation (e.g. how they were developed through group interactions), reflections on the meeting process (e.g. missed or misunderstood recommendations), challenges faced by staff in death preparation, and refined recommendations for preparing patients and families for an expected death. This framework guided the annotation, categorisation and synthesis of data under relevant headings, enabling refinement, elimination of duplicates and consolidation of related recommendations. During this process, the three tied recommendations missed in the final ranking were reintroduced and incorporated into the refined top-ranked recommendations, which included predicting life expectancy, delivering appropriate end-of-life care and supporting families’ psychological needs. Table 3 illustrates how this recommendation evolved into a more comprehensive form through analysis.

**Table 3.** Refining the top one recommendation from the nominal group meeting through framework analysis in the example study.

Charting stage of framework analysis							Refined recommendations after framework analysis
Recommendation generated from the meeting	Suggester	Ranking top 10	Discussion during the meeting	Reflections on the process of the meeting	Challenges previously experienced by staff	Refined recommendation after the charting stage	
*Clinical care-related dimension* **Healthcare staff can recognise dying signs and deliver care.**	Participant J	1^st^	Healthcare staff could recognise dying signs and alert family members that death was near, as family members aimed to accompany the patient during this critical period.	The listed strategy did not mention that it was crucial to alert family members to accompany the patient when death was near.	Missing the rationale	*Competencies* Healthcare staff should demonstrate competence in recognising dying signs, communicating effectively and timely with families about impending death, and delivering appropriate end-of-life care.	*Supplying tailored palliative and end-of-life care for patients, families, and significant others* • Healthcare staff should aim to predict life expectancy as accurately as possible, recognise dying signs, and deliver appropriate end-of-life care to patients and families. This includes: - Assist families in fulfilling the patient’s last wishes and unfinished business (*patients)* - Assist the patient in creating a legacy that can serve as a means of connecting with their families after death (*patients)* - Assist the patient in reviewing his/her life (*patients)* - Communicate effectively and timely with families about the impending death *(families)* - Guiding families to participate in the physical care of the patient *(families)* Recognising and addressing families’ psychological and emotional needs *(families)*

The final recommendations were developed from participants’ input and discussions, but their wording was modified for clarity and practical relevance. The final recommendations were not returned to participants for review or re-ranking due to no consent obtained for a follow-up.

Our study demonstrates the value of in-depth transcript analysis using an interpretive approach. Although the nominal group technique emphasises preserving participants’ original wording, this may limit the practical utility of recommendations. A more interpretive lens enhances clarity, feasibility and contextual relevance, while also providing deeper insights into the reasoning behind recommendations. It allows researchers to evaluate all recommendations within a broader context rather than focusing solely on the top ten generated during the meeting.

## Suggestions for supporting good practice in using the nominal group technique

The nominal group technique aims to build consensus by engaging practitioners in generating and prioritising recommendations.^5,9^ Based on our methodological reflections, we suggest several strategies for future research to strengthen its application and enhance recommendation quality (Table 4). These insights developed in palliative care research are relevant to other fields.

**Table 4.** Key suggestions for conducting nominal group techniques research.

Recommendations for researchers
**Conduct a pilot nominal group meeting**
• Pilot meetings should be conducted to pre-test research questions to be presented and meeting procedures. • Adjustments based on pilot findings should align with specific research needs and be clearly justified. • Pilot findings and their impact on the main meeting should be documented and reported. • Pilot meetings can serve as main meetings if no significant changes to the meeting procedures are required, and this should be planned (e.g. obtaining informed consent from participants).
**Define group consensus**
• Definition of group consensus should be clearly stated, including when predetermined agreement threshold met, such as through majority votes, ranking priorities or statistical measures, and when no significant objections remain.
**Present relevant evidence if necessary** • Whether to provide participants with relevant evidence on research topics during group meetings should be clearly justified to ensure transparency in the research process. If included, the evidence should be synthesised and presented to participants in a clear and succinct manner.
**Plan for practical challenges**
• Allocate appropriately sufficient time and have help from experienced researchers/moderators to overcome the practical challenges of conducting nominal group meetings posed by multiple tasks, time pressure, practical complexities and real-world uncertainties.
**Follow social norms in cultural contexts**
• Consider social norms, communication styles, and behaviours deemed appropriate in cultural contexts. • Allocate sufficient time, especially for conducting nominal group meetings in hierarchical societies emphasising politeness and respect for senior people.
**Analyse the rich qualitative data from the group meeting process**
• Review the conduct of nominal group meetings and reflect on the researcher’s role in these processes through additional in-depth qualitative data analysis, such as framework analysis. • Analyse the discussion during recommendation listing and clarification to gain deeper insights into the context and rationale behind participant-generated recommendations. • The final list of refined recommendations should be presented to participants for review.

Pilot meetings should be conducted to test research questions to be presented, refine meeting procedures, and report their impact on the main meeting. If no significant changes to the procedures are required, these pilot meetings can serve as main meetings, and data can be analysed to generate findings. This should be planned so that informed consent can be obtained. We also recommend that researchers clearly justify whether relevant evidence will be presented to participants during meetings to enhance transparency.

It is important to anticipate and address practical challenges when conducting group meetings, as strictly following the original plans is often unrealistic and may compromise the quality of recommendations. These challenges, often underreported in the literature, can persist even after pilot meetings due to multiple tasks, time constraints, practical complexities and real-world uncertainties. Such issues are particularly evident during the recommendation listing, clarification, voting and ranking stages. Cultural considerations are also essential when applying the nominal group technique. In societies where interruptions are avoided as a sign of respect and politeness, allocating sufficient time for group meetings, particularly during the recommendation listing and clarification stages, and using it efficiently is vital. Prior arrangements to accommodate participants’ communication styles can help ensure they fully express their recommendations.

Although time-consuming, in-depth analysis of meeting transcripts, particularly discussions during recommendation listing and clarification, using an interpretive qualitative approach is highly recommended for future studies. This approach allows for reflection on the group meeting process and the researcher’s role and can address practical challenges encountered during the process. It provides deeper insights into the context and rationale behind participant-generated recommendations, refining them for better applicability. To assess if the original consensus has changed, the final list of refined recommendations should be presented to participants for review, with the possibility of re-ranking the recommendations.

## Conclusion

The nominal group technique serves various research purposes and has significant potential to advance palliative care research and other fields. While methodologically structured, it remains flexible and can be adapted to specific research objectives or participant needs. Drawing from its theoretical foundations and practical applications in palliative care, we provide methodological reflections and identify key challenges: the absence of pilot meetings, neglecting practical challenges in conducting group meetings, insufficient attention to cultural factors and underutilisation of valuable data generated during meetings. These issues can undermine research rigour and the quality of recommendations. We also offer suggestions to address these methodological challenges to strengthen the application of the nominal group technique, including culturally appropriate approaches. This paper will support the development of more contextually relevant and implementable recommendations.

## Supplemental Material

sj-docx-1-pmj-10.1177_02692163251368974 – Supplemental material for Methodological reflections to support good practice in using nominal group techniques: Insights from applications in palliative care studies Supplemental material, sj-docx-1-pmj-10.1177_02692163251368974 for Methodological reflections to support good practice in using nominal group techniques: Insights from applications in palliative care studies by Hui-Ju Liang, Qian Xiong and Nancy Preston in Palliative Medicine

## References

[1] Platz T. Methods for the development of Healthcare practice recommendations using systematic reviews and meta-analyses. Front Neurol 2021; 12: 699968. 34305801 10.3389/fneur.2021.699968 PMC8297739

[2] Haines A Donald A. Making better use of research findings. BMJ 1998; 317: 72–75. 9651281 10.1136/bmj.317.7150.72 PMC1113463

[3] Curtis K Fry M Shaban RZ , et al Translating research findings to clinical nursing practice. J Clin Nurs 2017; 26: 862–872. 27649522 10.1111/jocn.13586 PMC5396371

[4] Voznyuk S Carter RZ Ridley J. A pragmatic approach to selecting a grading system for clinical practice recommendations in palliative care. Palliat Med 2025; 39: 176–185. 39369282 10.1177/02692163241286658 PMC11673308

[5] Jünger S Payne S. The crossover artist: consensus methods in health research. In: Walshe C Brearley S (eds) Handbook of theory and methods in applied health research: questions, methods and choices. UK: Edward Elgar, 2020, pp.199–213.

[6] Foth T Efstathiou N Vanderspank-Wright B , et al The use of Delphi and Nominal Group Technique in nursing education: a review. Int J Nurs Stud 2016; 60: 112–120. 27297373 10.1016/j.ijnurstu.2016.04.015

[7] Black N Murphy M Lamping D , et al Consensus development methods: a review of best practice in creating clinical guidelines. J Health Serv Res Policy 1999; 4: 236–248. 10623041 10.1177/135581969900400410

[8] Redman S Carrick S Cockburn J , et al Consulting about priorities for the NHMRC National Breast Cancer Centre: how good is the nominal group technique. Aust N Z J Public Health 1997; 21: 250–256. 9270149 10.1111/j.1467-842x.1997.tb01695.x

[9] Fink A Kosecoff J Chassin M , et al Consensus methods: characteristics and guidelines for use. Am J Public Health 1984; 74: 979–983. 6380323 10.2105/ajph.74.9.979 PMC1651783

[10] Delbecq AL Van de Ven AH. A group process model for problem identification and program planning. J Appl Behav Sci 1971; 7: 466–492.

[11] Harvey N Holmes CA. Nominal group technique: an effective method for obtaining group consensus. Int J Nurs Pract 2012; 18: 188–194. 22435983 10.1111/j.1440-172X.2012.02017.x

[12] Van de Ven AH Delbecq AL. The nominal group as a research instrument for exploratory health studies. Am J Public Health 1972; 62: 337–342. 5011164 10.2105/ajph.62.3.337 PMC1530096

[13] O. Nyumba T Wilson K Derrick CJ , et al The use of focus group discussion methodology: insights from two decades of application in conservation. Methods Ecol Evolut 2018; 9: 20–32.

[14] Wong LP. Focus group discussion: a tool for health and medical research. Singapore Med J 2008; 49: 256–260. 18363011

[15] Gallagher M Hares T Spencer J , et al The nominal group technique: a research tool for general practice? Fam Pract 1993; 10: 76–81. 8477899 10.1093/fampra/10.1.76

[16] Stevinson C Preston N Todd C , et al Defining priorities in prognostication research: results of a consensus workshop. Palliat Med 2010; 24: 462–468. 20501513 10.1177/0269216310368452

[17] Preston NJ Fayers P Walters SJ , et al Recommendations for managing missing data, attrition and response shift in palliative and end-of-life care research: part of the MORECare research method guidance on statistical issues. Palliat Med 2013; 27: 899–907. 23652842 10.1177/0269216313486952

[18] McMillan SS King M Tully MP. How to use the nominal group and Delphi techniques. Int J Clin Pharm 2016; 38: 655–662. 26846316 10.1007/s11096-016-0257-x PMC4909789

[19] Fox WM. The improved nominal group technique (INGT). J Manage Dev 1989; 8: 20–27.

[20] Coombes L Harðardóttir D Braybrook D , et al Achieving consensus on priority items for paediatric palliative care outcome measurement: results from a modified Delphi survey, engagement with a children’s research involvement group and expert item generation. Palliat Med 2023; 37: 1509–1519. 37853579 10.1177/02692163231205126 PMC10657511

[21] Walshe C Dunleavy L Preston N , et al Understanding barriers and facilitators to palliative and end-of-life care research: a mixed method study of generalist and specialist health, social care, and research professionals. BMC Palliat Care 2024; 23: 159. 38918771 10.1186/s12904-024-01488-2 PMC11202245

[22] Rice DB Cañedo-Ayala M Turner KA , et al Use of the nominal group technique to identify stakeholder priorities and inform survey development: an example with informal caregivers of people with scleroderma. BMJ Open 2018; 8: e019726. 10.1136/bmjopen-2017-019726 PMC5855214 29500214

[23] Hökkä M Ravelin T Coupez V , et al Core palliative care competencies for undergraduate nursing education: international multisite research using online nominal group technique. J Palliat Care 2024; 39: 217–226. 38584432 10.1177/08258597241244605 PMC11097607

[24] Higginson IJ Evans CJ Grande G , et al Evaluating complex interventions in end of life care: the MORECare statement on good practice generated by a synthesis of transparent expert consultations and systematic reviews. BMC Med 2013; 11: 1–11. 23618406 10.1186/1741-7015-11-111 PMC3635872

[25] Harrop E Liossi C Jamieson L , et al Oral morphine versus transmucosal diamorphine for breakthrough pain in children: methods and outcomes: UK (DIPPER study) consensus. BMJ Support Palliat Care 2023; 13: e1019–e1028. 10.1136/bmjspcare-2021-003278 PMC10850727 34903585

[26] Hussain JA White IR Johnson MJ , et al Development of guidelines to reduce, handle and report missing data in palliative care trials: a multi-stakeholder modified nominal group technique. Palliat Med 2022; 36: 59–70. 35034529 10.1177/02692163211065597 PMC8796167

[27] Dhingra L Braveman C Roberts K , et al Low hospice utilization in New York State: framework for compiling and ranking barriers. J Palliat Med 2022; 25: 1524–1532. 35417252 10.1089/jpm.2022.0004

[28] Walshe C Kinley J Patel S , et al A four-stage process for intervention description and guide development of a practice-based intervention: refining the Namaste Care intervention implementation specification for people with advanced dementia prior to a feasibility cluster randomised trial. BMC Geriatr 2019; 19: 1–11. 31638902 10.1186/s12877-019-1275-z PMC6802319

[29] de Wolf-Linder S Dawkins M Wicks F , et al Which outcome domains are important in palliative care and when? An international expert consensus workshop, using the nominal group technique. Palliat Med 2019; 33: 1058–1068. 31185812 10.1177/0269216319854154 PMC6691595

[30] Tuffrey-Wijne I Wicki M Heslop P , et al Developing research priorities for palliative care of people with intellectual disabilities in Europe: a consultation process using nominal group technique. BMC palliative care 2016; 15: 1-11. 27009550 10.1186/s12904-016-0108-5 PMC4806426

[31] van Riet Paap J Vissers K Iliffe S , et al Strategies to implement evidence into practice to improve palliative care: recommendations of a nominal group approach with expert opinion leaders. BMC Palliat Care 2015; 14: 1–6. 26419434 10.1186/s12904-015-0044-9 PMC4589187

[32] Dening KH Jones L Sampson EL. Preferences for end-of-life care: a nominal group study of people with dementia and their family carers. Palliat Med 2013; 27: 409–417. 23128905 10.1177/0269216312464094 PMC3652642

[33] Pastrana T Radbruch L Nauck F , et al Outcome indicators in palliative care—how to assess quality and success. Focus group and nominal group technique in Germany. Support Care Cancer 2010; 18: 859–868. 19701782 10.1007/s00520-009-0721-4 PMC3128732

[34] Tuffrey-Wijne I Bernal J Butler G , et al Using Nominal Group Technique to investigate the views of people with intellectual disabilities on end-of-life care provision. J Adv Nurs 2007; 58: 80–89. 17394619 10.1111/j.1365-2648.2007.04227.x

[35] Aspinal F Hughes R Dunckley M , et al What is important to measure in the last months and weeks of life?: A modified nominal group study. Int J Nurs Stud 2006; 43: 393–403. 16102767 10.1016/j.ijnurstu.2005.06.005

[36] Liang H-J Xiong Q Lin P-C , et al ‘A good ending but not the end’: exploring family preparations surrounding a relative’s death and the Afterlife–a qualitative study. Palliat Med 2024; 38(10): 1184–1193. 39340161 10.1177/02692163241280016 PMC11613525

[37] Liang H-J Xiong Q Lin P-C , et al ‘Regrets become a lasting source of pain’: a qualitative study on family caregivers’ experiences leading up to a relative’s death. Palliat Med 2025; 39(3): 401–412. 39927610 10.1177/02692163251316677 PMC11877984

[38] Liang H-J Xiong Q Remawi BN , et al Taiwanese family members’ bereavement experience following an expected death: a systematic review and narrative synthesis. BMC Palliat Care 2024; 23: 14. 38212776 10.1186/s12904-024-01344-3 PMC10782629

[39] McEvoy P Richards D. A critical realist rationale for using a combination of quantitative and qualitative methods. J Res Nurs 2016; 11: 66–78.

[40] Carney O McIntosh J Worth A. The use of the nominal group technique in research with community nurses. J Adv Nurs 1996; 23: 1024–1029. 8732532 10.1046/j.1365-2648.1996.09623.x

[41] Evans CJ Benalia H Preston NJ , et al The selection and use of outcome measures in palliative and end-of-life care research: the MORECare international consensus workshop. J Pain Symptom Manage 2013; 46: 925–937. 23628515 10.1016/j.jpainsymman.2013.01.010 PMC3858887

[42] Gysels M Evans CJ Lewis P , et al MORECare research methods guidance development: recommendations for ethical issues in palliative and end-of-life care research. Palliat Med 2013; 27: 908–917. 23695828 10.1177/0269216313488018

[43] McMillan SS Kelly F Sav A , et al Using the nominal group technique: how to analyse across multiple groups. Health Serv Outcomes Res Methodol 2014; 14: 92–108. 10.1186/1472-6963-14-476 PMC4282516 25281284

[44] Yum JO. The impact of Confucianism on interpersonal relationships and communication patterns in East Asia. Commun Monogr 1988; 55: 374–388.

[45] Mullen R Kydd A Fleming A , et al A practical guide to the systematic application of nominal group technique. Nurse Res 2021; 29: 14–20. 10.7748/nr.2021.e1777 33629547

[46] Parkinson S Eatough V Holmes J , et al Framework analysis: a worked example of a study exploring young people’s experiences of depression. Qual Res Psychol 2016; 13: 109–129.

[47] Ritchie J Spencer L . Qualitative data analysis for applied policy research. In: Bryman A Burgess RG (eds) Analyzing qualitative data. UK: Routledge, 2002, pp.173–194.

