# Supplementary material for: Methodological reflections to support good practice in using nominal group techniques: Insights from applications in palliative care studies
Source: Palliat Med. 2025 Sep 28;39(10):1099–108. doi: 10.1177/02692163251368974 (PMC12640363; doi:10.1177/02692163251368974)
Supplement: sj-docx-1-pmj-10.1177_02692163251368974 – Supplemental material for Methodological reflections to support good practice in using nominal group techniques: Insights from applications in palliative care studies [file sj-docx-1-pmj-10.1177_02692163251368974.docx]

**Supplementary file (S1)**: A summary of exemplar palliative and end-of-life studies using the nominal group technique (N=16)

| Authors, year, country | Aim/purpose | NG meeting/  No. of NG | Attendees of NG meeting/Pilot | Definition of consensus | Post-meeting analysis | Challenges encountered in practice |
| --- | --- | --- | --- | --- | --- | --- |
| Aspinal et al., 2006, United Kingdom | Explore key aspects to measure at end of life for patients, bereaved relatives, and healthcare professionals’ perspectives | Two-hour in-person NG (10): 4 professionals,4 relatives  2 patients | 75 participants: 10 patients with cancer, 35 professionals & 30 bereaved relatives  No pilot mentioned | Top five ranked items | Descriptive statistical analysis: total and median rating score for each item, how many times ranked 1st to 5th  Thematic analysis: statements generated from groups | *Participant- related & conduct of meeting*  Same research question to all participant groups (recognised might be interpreted differently)  *Data analysis*  Analysing across groups challenging, particularly combining voting and ranking results to establish an overall consensus across groups |
| Tuffrey‐Wijne et al., 2007, United Kingdom | Gather perspectives on end-of-life care provision | Two-hour in-person NG (3) | 14 people with intellectual disabilities  No pilot mentioned | Top five ranked important ideas | Descriptive statistical analysis: total score for ranking results  No qualitative analysis | *Participant-related & conduct of meeting*  Challenges in grouping, sorting and ranking recommendations for participants with limited literacy  *Data analysis*  Analysing across groups challenging, particularly recommendations generated across groups |
| Pastrana et al., 2010, Germany | Explore key indicators for evaluating palliative care quality | In-person NG (1)  Unclear duration | 9 experts from multiple disciplines  No pilot mentioned | Higher- ranked indicator scores | Descriptive statistical analysis: total score for ranking results  Thematic analysis: meeting transcripts | *Establish consensus*  Diverse recommendations made consensus difficult |
| Stevinson et al., 2010, United Kingdom | Elicit and rank research questions to reach consensus on priorities for prognostication research | In-person workshop with NG (4)  Unclear duration | 25 delegates: 10 palliative care physicians, 4 nurses, 1 dietician & 10 academic researchers  No pilot mentioned | Top ten ranked research questions | Descriptive statistical analysis: voting and ranking results  No qualitative analysis | Not addressed |
| Dening et al., 2013, United Kingdom | Explore preferences for end-of-life care | 55-minute in-person NG (3): 1 persons with dementia, 1 carers & 1 dyads of people with dementia and carers | 17 participants: 9 people with dementia, 8 carers  No pilot mentioned | Top five ranked important ideas items | Descriptive statistical analysis: ranking scores from 3 NGs to determine the overall item priorities Qualitative content analysis: meeting transcripts | Not addressed |
| Higginson et al., 2013, United Kingdom | Develop evidence-based guidance on optimal research design and conduct for end-of-life care research | One-day in-person workshop with NG (3) for five topics* | 140 participants  No pilot mentioned | Higher levels of agreement | Descriptive statistical analysis: median score, interquartile range  No specific method: participant-generated recommendations,  Scribe notes, meeting transcripts | *Participant- related*  Limited participant diversity with only conference attendees invited  *Conduct of meeting*  Limited available time, especially for discussion  *Data analysis*  Analysing multi-part recommendations that had multiple parts, balancing simplification with preserving original wording |
| van Riet Paap et al., 2015, Spain | Develop recommendations for integrating evidence into palliative care  practice | In-person NG (2)  Unclear duration | 20 participants: 11 researchers & 9 clinicians  No pilot mentioned | Top five ranked items | Descriptive statistical analysis: ranking results  No specific method: all items mentioned by participants | Not addressed |
| Tuffrey-Wijne et al., 2016, Switzerland | Develop research priorities for palliative care of people with intellectual disabilities | Two-hour in-person NG (1) | 12 academic & clinicians  No pilot mentioned | Top five ranked  important research themes | Not stated | Not addressed |
| Rice et al., 2018, Canada and United States | Develop survey items to assess challenges and for support service preferences | In-person NG (3): 2 in USA & 1 in Canada  Duration: 90-120 minutes | 13 informal caregivers of individuals with systematic sclerosis  Pilot conducted | Top ten ranked  challenges and support services | Descriptive statistical analysis: mean scores  Qualitative content analysis: all generated items | *Participant-related*  Limited participant diversity with only family caregivers at national patient conferences included |
| de Wolf-Linder et al., 2019, Ireland | Identify key palliative care domains for outcomes measurement and determine the optimal assessment timeframe | 90-minute in-person workshop with NG (4) | 33 clinicians & researchers  No pilot mentioned | Top five ranked outcome domains | Descriptive statistical analysis: mean score  Thematic analysis: scribe notes | *Participant-related*  Limited participant diversity (e.g., lack of clinical frontline staff, such as chaplaincy) |
| Walshe et al., 2019, United Kingdom | Refine and prioritise the Namaste Care programme implementation process | One-day in-person NG (2) | 17 participants: 15 nursing care home staff, 1 family carer & 1 volunteer  No pilot mentioned | Agreement on summary of NG results | Descriptive statistical analysis: frequency of rankings  Thematic analysis: meeting notes, participant-generated ideas | *Participant-related*  Ensuring geographical diversity among participants  Recruiting non-healthcare professionals for the study (e.g., family carers & volunteers) |
| Dhingra et al., 2022, United States | Identify and rank barriers to hospice utilisation | 90-minute in-person NG (6) | 57 stakeholders: 10 clinicians, 41 administrators & 6 others (e.g., professor)  No pilot mentioned | Top five ranked important barriers | Descriptive statistical analysis: voting results.  Thematic analysis: meeting transcripts | *Participant-related*  Limited participant diversity (e.g., lack of government workers) |
| Hussain et al., 2022, United Kingdom | Develop guidelines for addressing missing data in palliative care clinical trials | One-day in-person workshop with NG (5) | 39 delegates (e.g., patient and public research partners, clinicians & researcher)  No pilot mentioned | Top three ranked items | Descriptive statistical analysis: frequency of votes  Thematic analysis: scribe notes | *Participant-related*  Limited participant diversity (e.g., lack of geographical representation)  *Participant rechecking*  Final recommendations not presented to participants to review or re-scoring |
| Harrop et al., 2023, United Kingdom | Reach consensus on pain outcome measures and best practices for administering and dosing transmucosal diamorphine in breakthrough pain for children and young people receiving palliative care | In-person NG (1)  Unclear duration | 13 participants: 10 clinicians & 3 parent representatives  No pilot mentioned | Agreement on voting and ranking results was reached, with no further comments from participants | Descriptive statistical analysis: voting results  Thematic analysis: meeting transcript | Not addressed |
| Hökkä et al., 2024, Austria, Belgium, Finland, Romania | Identify and assess core palliative care competencies that European nurses need to attain in their education to deliver palliative care | Two-hour online NG (4) | 35 palliative care experts  Pilot conducted | Top ten tanked responses | Grouping top ten ranked responses across groups  Thematic analysis: recommendations that appeared in at least two groups across four meetings | *Participant rechecking*  Findings not presented to participants for feedback |
| Walshe et al., 2024, United Kingdom | Explore the professional and organisational factors that facilitate or hinder palliative and end-of-life care research | Two-hour online NG (4) | 20 participants: 9 nurses, 5 doctors, 3 managers/  administrative staff & 3 from other roles  No pilot mentioned | Top ten tanked ideas | Summarise key issues from the group transcripts using ranking results as an initial analytical framework | *Participant-related*  Recruiting the public for the study |

NG: nominal group meeting. * This was a transparent expert consultation involving nominal groups and follow-up online surveys.
